# Supplementary material for: A generalized Stark effect electromodulation model for extracting excitonic properties in organic semiconductors
Source: Nat Commun. 2019 Nov 8;10:5089. doi: 10.1038/s41467-019-13081-w (PMC6841700; doi:10.1038/s41467-019-13081-w)
Supplement: Supplementary file 1 — Supplementary Information [file 41467_2019_13081_MOESM1_ESM.pdf]

Supplementary Information:

A Generalized Electromodulation Model to Extract the Excitonic Properties in Organic Semiconductors

Liu *et al.*

Supplementary Figures

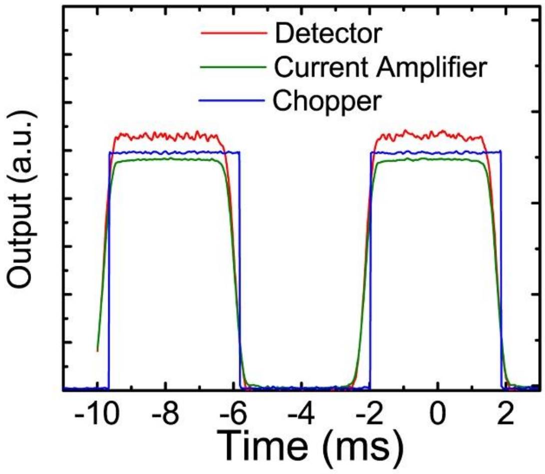

Supplementary Figure 1. Oscilloscope display of output signal. The red line depicts time response of detector, the green line stands for response of current amplifier and the blue line represents the response of chopper reference.

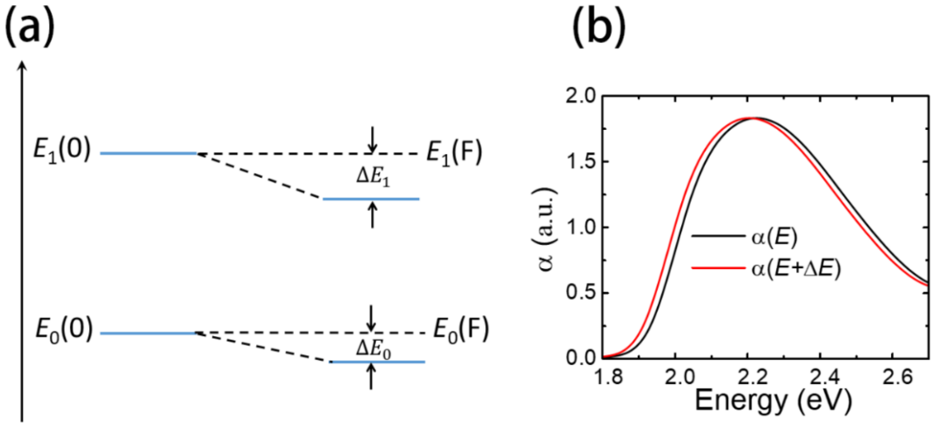

Supplementary Figure 2. Description of absorption red shift. (a) Energy diagram without and with electric field. E_0 (0) and E_0 (F) respectively represents ground state energy level without and with electric field, respectively. E_1 (0) and E_1 (F) respectively represents excited state energy level without and with electric field, respectively. ΔE_1 and ΔE_0 respectively stands for excited state and ground state energy shift. The Y-axis on the left is used to define the positive direction or increasing direction of energy. (b)

Absorption coefficient spectrum without electric field (dark line) and with electric field (red line).

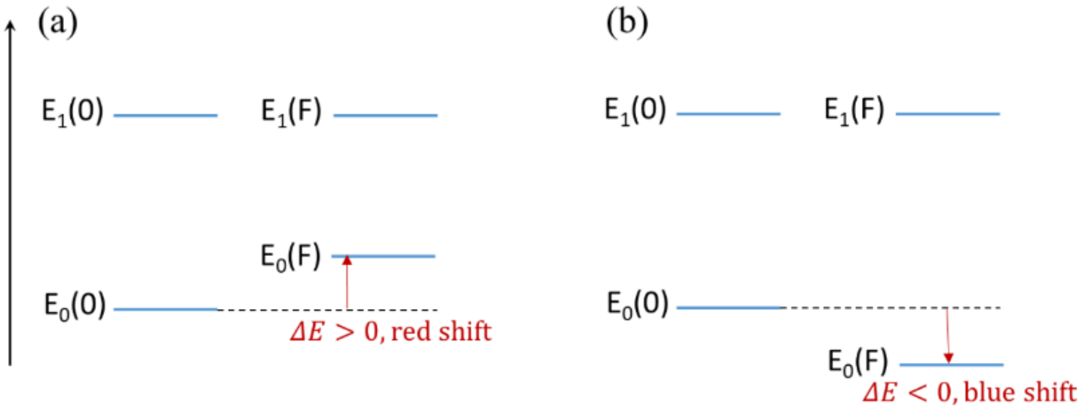

Supplementary Figure 3. Energy level shift due to Stark effect. (a) Red shift case. (b) Blue shift case.

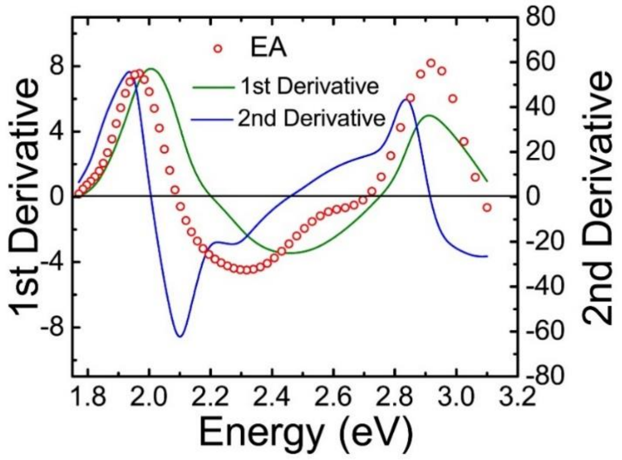

Supplementary Figure 4. Electroabsorption signal versus 1st and 2nd derivatives. Electroabsorption signal is shown as red circles and derivatives are shown as lines.

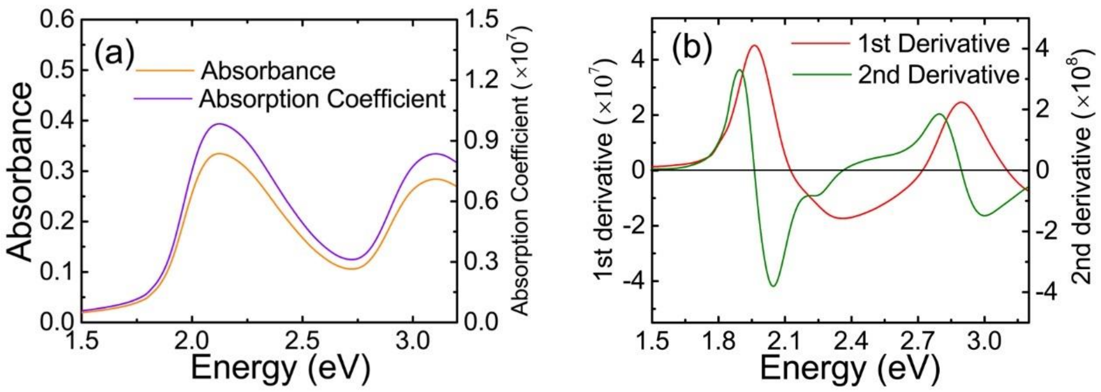

Supplementary Figure 5. PCDTBT thin film absorption profile and its derivatives. (a) Thin film absorbance and absorption coefficient calculated from thin film absorbance over its thickness. (b) The corresponding derivatives.

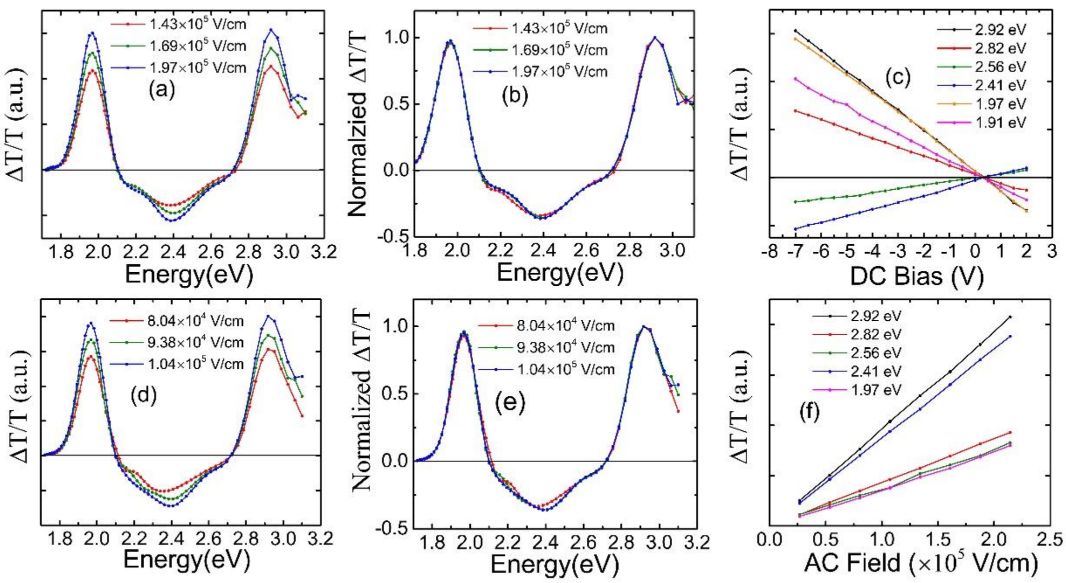

Supplementary Figure 6. Electric field dependence of the electromodulation signal. (a) Electromodulation (EM) DC field dependence. (b) Normalized EM DC field dependence which shows no obvious spectral change. (c) DC field dependence at certain photon energy which shows linear feature. (d) EM AC field dependence. (e) Normalized EM AC field dependence which shows no obvious spectral shift or broadening. (f) AC field dependence at certain photon energy which shows linear feature. The DC and AC field strength of all our EM experiment are within the range of above ones.

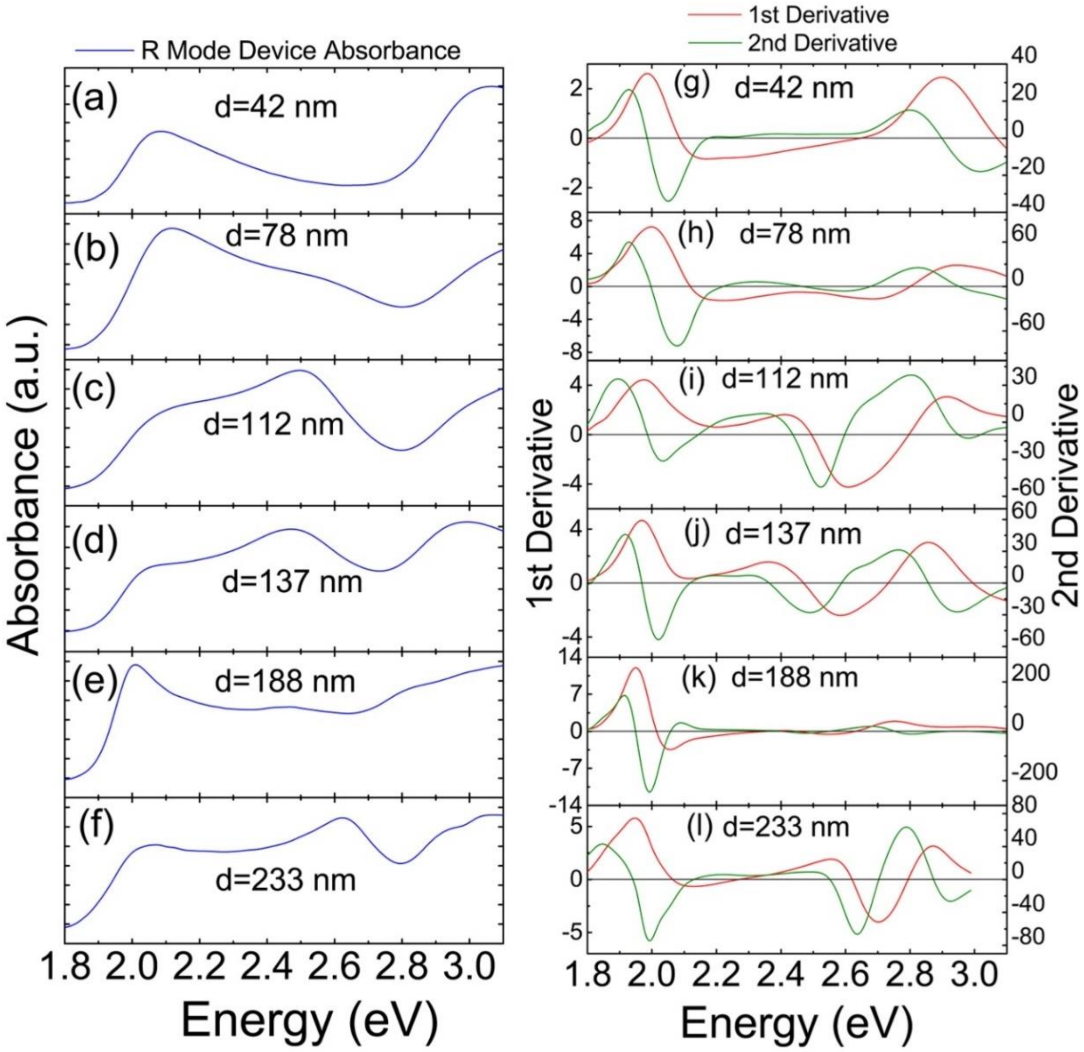

Supplementary Figure 7. Reflection mode device absorbance profile and derivatives. (a)-(f) Reflection (R) mode device absorbance. (g)-(l) 1st derivative and 2nd derivative of R mode device absorbance of corresponding thickness.

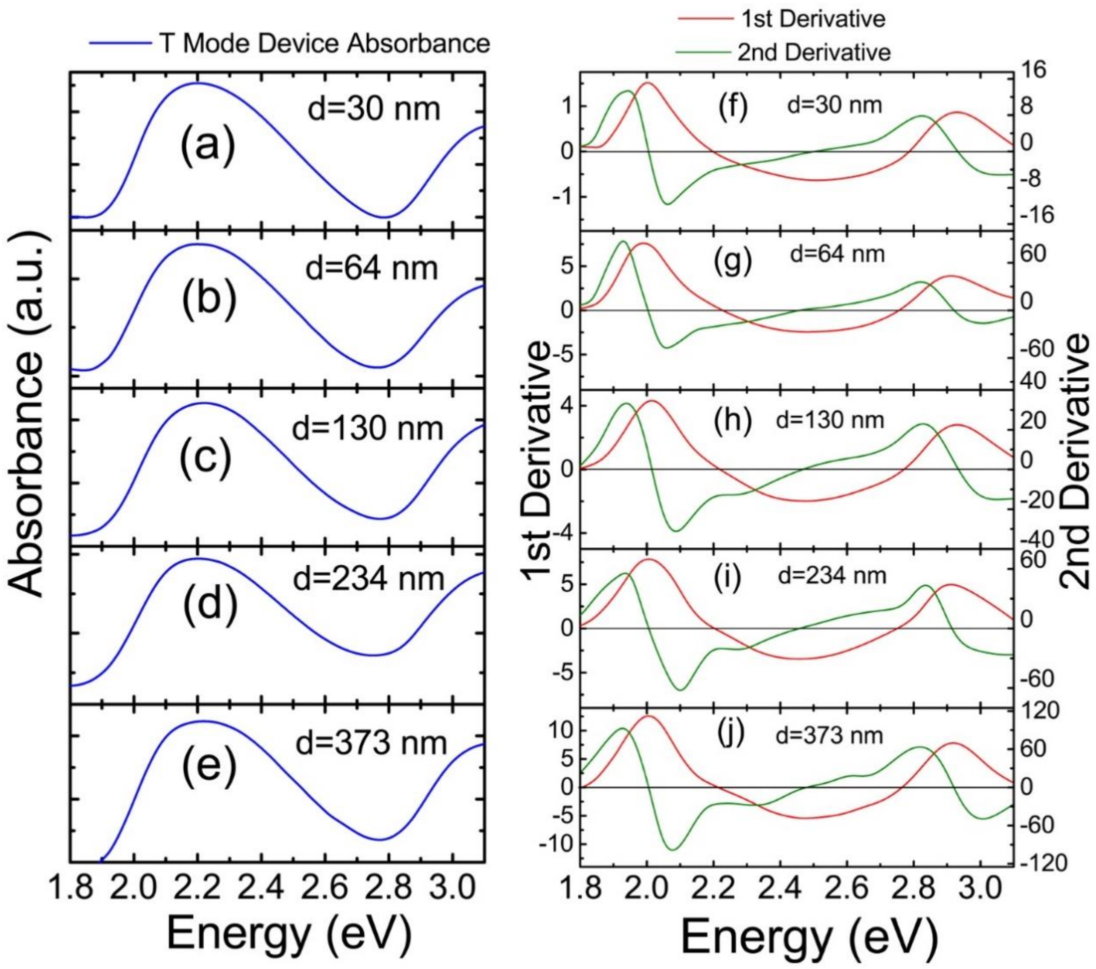

Supplementary Figure 8. Transmission mode device absorbance profile and derivatives. (a)-(e) Transmission (T) mode device absorbance. (f)-(j) 1st derivative and 2nd derivative of T mode device absorbance of corresponding thickness.

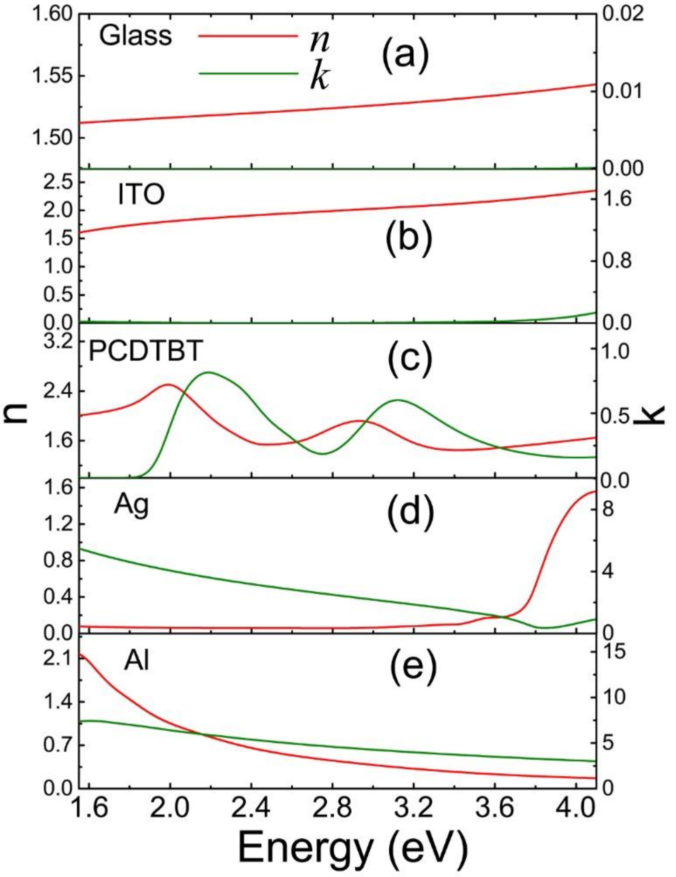

Supplementary Figure 9. *n* and *k* value of each layer used in device. (a) Glass substrate. (b) ITO. (c) PCDTBT. (d) Ag and (e) Al.

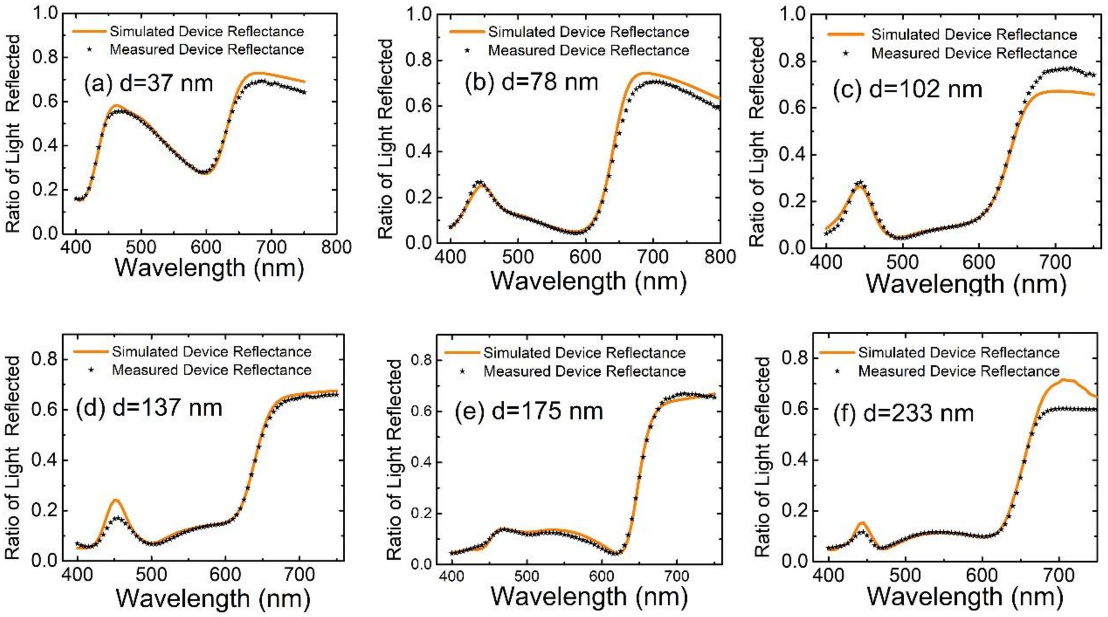

Supplementary Figure 10. Comparison of device transmission ratio with simulated one. (a)-(f) Reflection mode optical simulation (orange line) which shows good agreement with experimental small angle device reflectance.

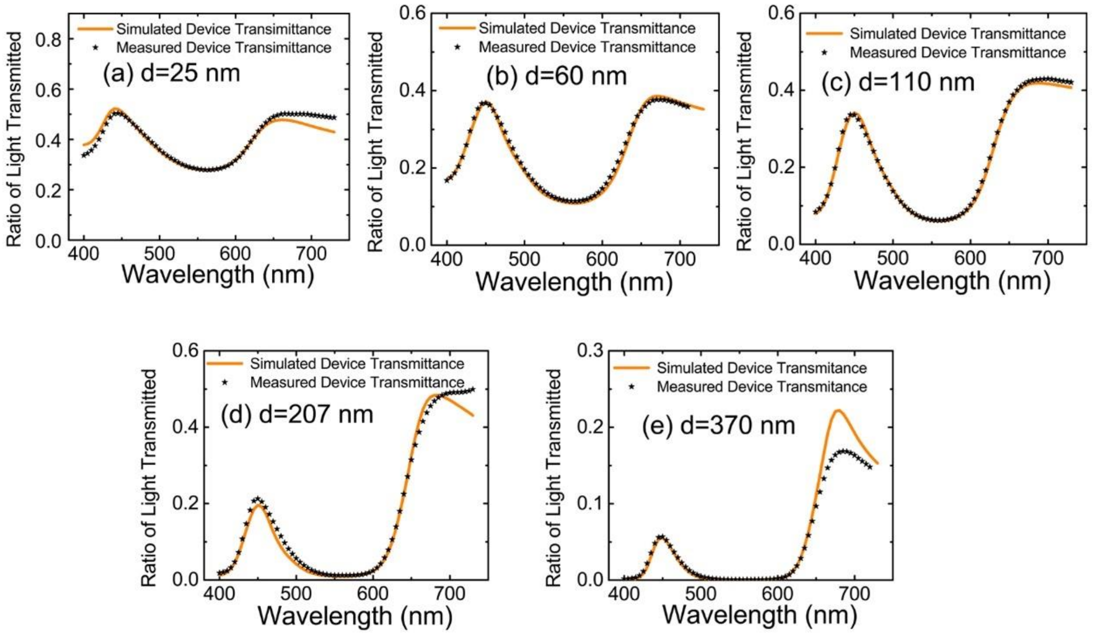

Supplementary Figure 11. Comparison of device reflection ratio with simulated one. (a)-(e) Transmission mode optical simulation (orange line) which shows good agreement with experimental normal incident angle device transmittance.

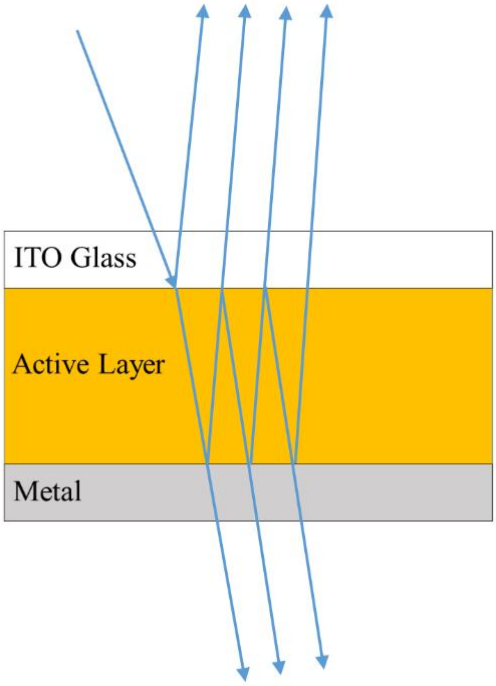

Supplementary Figure 12. Multiple reflection in device. For simplicity, we mainly consider reflection at ITO glass/active layer interface and multiple reflection within the active layer.

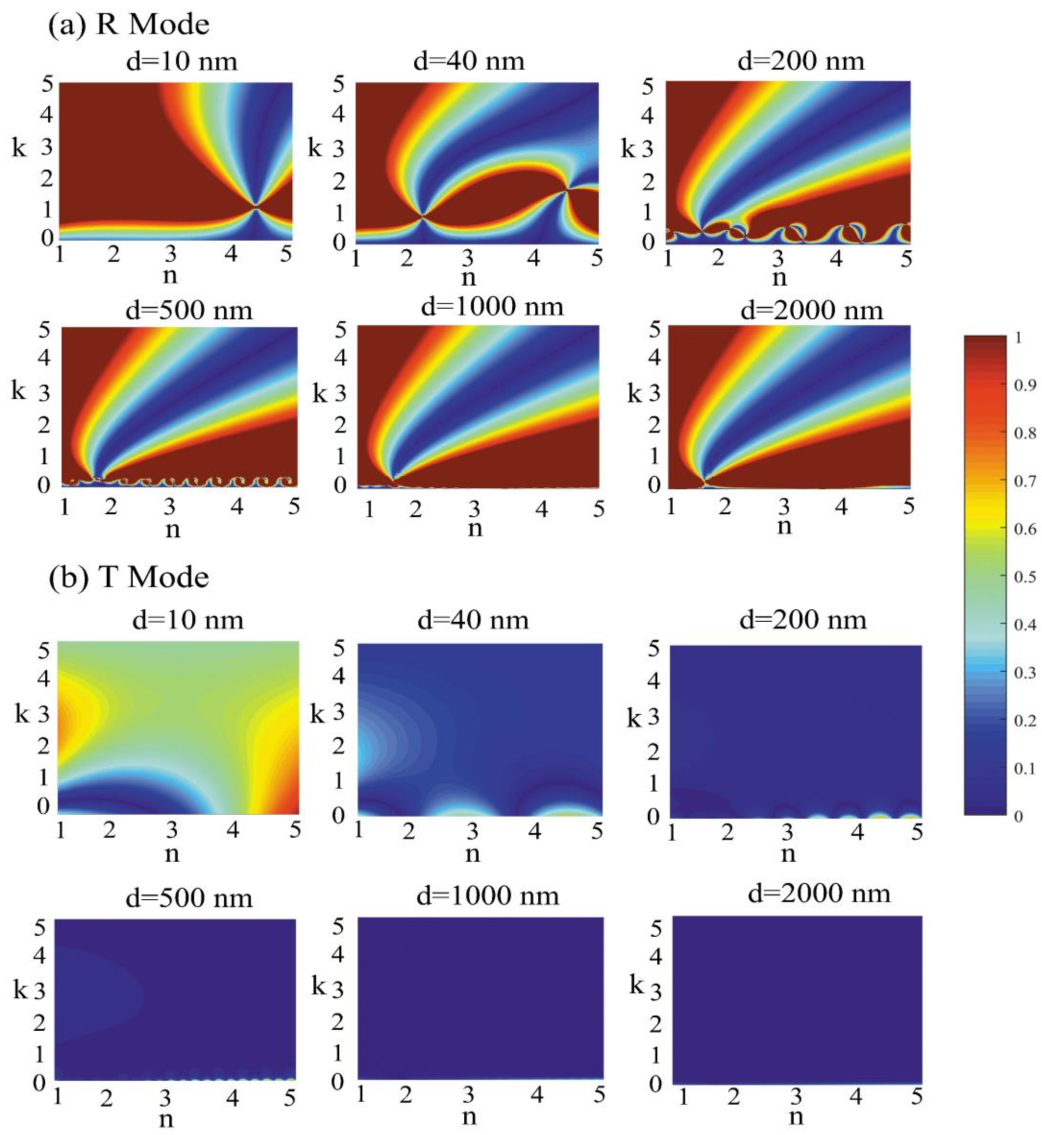

Supplementary Figure 13. Contour plot of $\frac{\partial I}{\partial n}$:$\frac{\partial I}{\partial k}$. (a) Reflection mode. (b) Transmission mode. Excitation wavelength: 400nm. Device configuration is ITO (110 nm)/active layer (d nm) /Ag (16 nm). The bar on the right depicts the value of the ratio which is fixed in the range of 0~1, and in the highest intensity region the ratio is no less than 1.

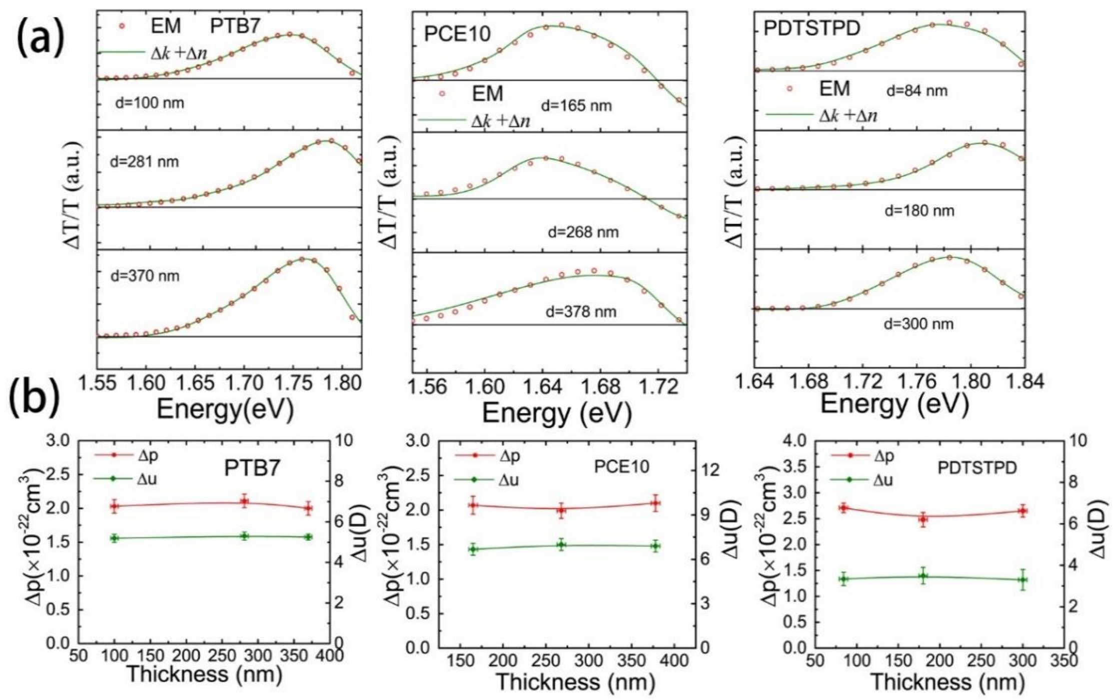

Supplementary Figure 14. Thickness dependent fitting results for 3 different materials. (a) Fitting curves of PTB7, PCE10 and PDSTPD with different thicknesses in transmission mode using the generalized electromodulation model. (b) Fitting values (Δp and Δu) vs thickness of the three different materials.

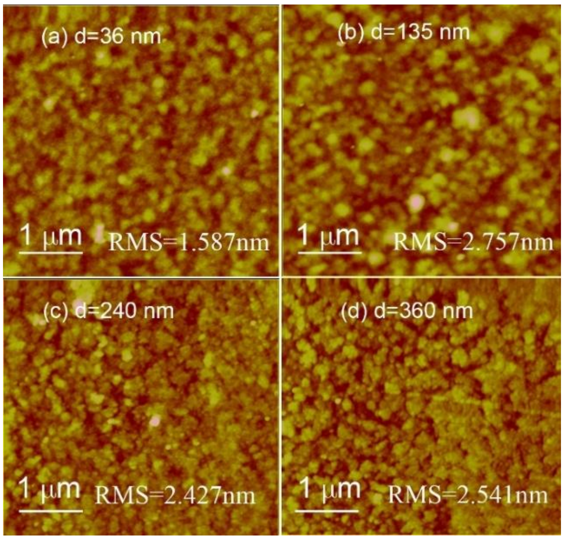

Supplementary Figure 15. AFM images of PCDTBT films on ITO with thickness. (a) 36 nm. (b) 135 nm. (c) 240 nm and (d) 360 nm. The RMS roughness of all films are less than 3 nm.

Supplementary Notes

Supplementary Note 1. $+\frac{\Delta I}{I}$ Or $-\frac{\Delta I}{I}$?

In previous reports, it is found that the sign used for $\frac{\Delta I}{I}$ is confusing, it can be sometimes positive [1-3]or negative [4-6] or even can be both in the same work[7, 8]. We sought to understand this issue by re-visiting the experimental and theoretical approach in the EM measurement.

In the Stark effect EM measurement, the light intensity passing through the sample is being modulated by an AC electrical field across the sample. Therefore, the measured light intensity $I[F(t)]$ is also a function of both electrical field strength F and time t. Since the sign of $\frac{\Delta I}{I}$ is determined by the phase difference measured by the lock-in amplifier between the reference signal (from light chopper or function generator) and the output signal from the current amplifier, it should firstly be verified if there exist a phase change in the output of the current amplifier $V[F(t)]$ after the input light intensity $I[F(t)]$ passing through the detector and current amplifier . As shown in Supplementary Figure 1, we measured the temporal signal from both chopper reference, detector, and current amplifier by a digital oscilloscope, the blue line is the reference signal from light chopper, the green line is voltage signal output by the current amplifier which reflects the chopped light intensity, and the red line is output signal from the detector. During the measurement, the frequency filter was turned off and the gain was set to be 20uA/V in the current amplifier, the same as in our EM measurement. It can be seen that those signals are synchronized with negligible phase difference. The above

discussion demonstrates that there is no additional phase change induced by the measurement setup.

Now we discuss how the sign of $\frac{\Delta I}{I}$ should be chosen to represent the measured EM results. It should be noted that the sign of measured $\frac{\Delta I}{I}$ is dependent on the sign of the DC electrical field across the organic layer, as demonstrated in Supplementary Figure 6(c). It is because when the AC electrical field superimposed with the DC, the resulting electrical field with respect to the AC reference signal from the function generator might have 180° phase difference. In case of an AC superimposed with a positive DC voltage is applied, the overall output voltage is in phase with the reference signal. However, in case of an AC superimposed with a negative DC voltage is applied, the overall output voltage is 180° phase different from the reference signal. For example, the all the results shown in this work were measured with a negative biased DC voltage. The measured EM results are positive at the 1st excitonic position while using a positive sign of $\frac{\Delta I}{I}$. It means that the light intensity increases with reduced electrical field (the overall electrical field is 180° out of phase with the reference signal). This is consistent with the Stark effect that the absorption spectrum red-shifted under an electrical field as commonly found in organic materials, as illustrated in Supplementary Figure 2(b).

Therefore, in order to resolve the confusion in presenting the measured EM data, it is suggested that both the sign of $\frac{\Delta I}{I}$ and the sign of the applied DC electrical field from the function generator should be clearly presented.

Supplementary Note 2. Derivation of fitting equation for isotropic materials

The fitting equation of electroabsorption to correlate the Stark effect and measured modulated light intensity was derived by L. Sebastain et al [9]. Briefly, the change of absorption coefficient $\Delta\alpha$ of a thin film is approximated in terms of Taylor series truncated at the quadratic term, i.e.

$$\Delta\alpha = \alpha(E+\Delta E) - \alpha(E) \approx \frac{\partial\alpha}{\partial E}\Delta E + \frac{1}{2}\frac{\partial^2\alpha}{\partial E^2}\Delta E^2 \tag{1}$$

As illustrated in Supplementary Figure 2(a), the key to deriving the fitting equation depends on the approach to define the overall change of transition energy ΔE, due to energy level shift in both ground state $\Delta E_0 = E_0(F) - E_0(0)$ and excited state $\Delta E_1 = E_1(F) - E_1(0)$ under an electrical field *F*. L. Sebastain et al[9] proposed the definition of ΔE as excited state energy level change minus ground state energy level change, i.e.,

$$\Delta E \equiv \Delta E_1 - \Delta E_0 \tag{2}$$

By applying Equation (2) in the manuscript to the above Equation (2), then we have

$$\begin{aligned} \Delta E &= [E_1(F) - E_1(0)] - [E_0(F) - E_0(0)] \\ &= -u_{1z}\cdot F - \frac{1}{2}p_1\cdot F^2 - \left(-u_{0z}\cdot F - \frac{1}{2}p_0\cdot F^2\right) \\ &= -(u_{1z} - u_{0z})\cdot F - \frac{1}{2}(p_1 - p_0)\cdot F^2 \\ &= -\Delta u_z\cdot F - \frac{1}{2}\Delta p\cdot F^2 \end{aligned} \tag{3}$$

Therefore, the change of absorption coefficient can be expressed by combing Equation (3) with Equation (1), i.e.

$$\Delta\alpha \approx \left[-\Delta u_z F \frac{\partial\alpha}{\partial E} - \frac{1}{2}\Delta p F^2 \frac{\partial\alpha}{\partial E}\right] + \frac{1}{2}\left(-\Delta u_z \cdot F - \frac{1}{2}\Delta p \cdot F^2\right)^2 \frac{\partial^2\alpha}{\partial E^2} \tag{4}$$

It is worth to be noted that Equation (1) describes the case for a single molecule, therefore ΔE in Equation (2) and $\Delta\alpha$ in Equation (4) also represent the change of energy level and absorption coefficient of a single molecule, respectively. In case of a bulk film, the change of absorption coefficient is an isotropic average over all orientations of the dipoles versus the external electrical field. Thus, $\Delta u_z = \pm|\Delta u_z|$, the change of dipole moment along the electrical field can be positive or negative with equal probability, so the average value of the term $-\Delta u_z F \frac{\partial\alpha}{\partial E}$ in the first bracket of Equation (4) vanishes. For the second bracket in Equation (4), we approximate to the second order dependence of electrical field, so that we have the change of absorption coefficient with respect to the quadratic Stark effect, i.e.

$$\Delta\alpha \approx -\frac{1}{2}\Delta p F^2 \frac{\partial\alpha}{\partial E} + \frac{1}{2}(\Delta u_z \cdot F)^2 \frac{\partial^2\alpha}{\partial E^2} \tag{5}$$

Now Δp in Equation (5) is the macroscopic average value of polarizability change for all orientations. Meanwhile, the component of the permanent dipole moment in any direction is the same in magnitude for an isotropic material, thus $\Delta u_x^2 = \Delta u_y^2 = \Delta u_z^2 = \frac{1}{3}\left(\Delta u_x^2 + \Delta u_y^2 + \Delta u_z^2\right) = \frac{1}{3}\Delta u^2$, then Equation (5) can be expressed as

$$\Delta\alpha \approx -\frac{1}{2}\Delta p F^2 \frac{\partial \alpha}{\partial E} + \frac{1}{6}(\Delta u \cdot F)^2 \frac{\partial^2 \alpha}{\partial E^2} \quad (6)$$

Δu in Equation (6) is the macroscopic average value of permanent dipole moment change for all orientations. Therefore, the change in optical absorption $\Delta\alpha$ in thin film can be explicitly expressed as a function of changes of average polarizability Δp and average permanent dipole moment Δu of the material upon photoexcitation from ground state to the excited state. $\frac{\partial \alpha}{\partial E}$ and $\frac{\partial^2 \alpha}{\partial E^2}$ are the 1st and 2nd derivatives of the optical absorption of the material, and F is the electrical field.

The definition of ΔE shown in Equation (2) and Equation (3) together with fitting Equation (6) has been widely adapted in previous reports [4, 5, 10-14]. However, we have noted that in several reports when using Equation (6) for fitting with the experimental results, the sign before the first term on the right-hand side has been changed to positive [7, 15, 16]. In fact, we have discovered that ΔE as defined in Equation (2) results in contradiction between the assumption and conclusion. As shown in Supplementary Figure 2 (a) and (b), without the loss of generality, considering that both the ground and excited states are shifted downward with an overall of reduced energy gap under an electric field, which corresponds to a red shift of α. For instance, if $\Delta E_1 = -0.05\text{eV}$ and $\Delta E_0 = -0.01\text{eV}$, using $\Delta E \equiv \Delta E_1 - \Delta E_0$, then $\Delta E = -0.04eV < 0$. As a result, the absorption coefficient under an electrical field $\alpha(E + \Delta E)$ is blue shifted relative to $\alpha(E)$ and it contradicts with the initial assumption of red shift with reduced energy gap. On the ther hand, if ΔE is defined as $\Delta E \equiv \Delta E_0 - \Delta E_1$, then

$\Delta E = 0.04$, and $\alpha(E+\Delta E)$ is red shifted which coincides with the assumption. In fact, the physical meaning of $\Delta E \equiv \Delta E_0 - \Delta E_1$ can be regareded as the relative change of energy level E_0 to E_1 under electric field. As is demonstrated in Supplementary Figure 3. In this case, E_1 is treated as static and only the change of E_0 is considered. Therefore, for $\Delta E > 0$, E_0 will move towards to E_1 which corresponds to a red shift; for $\Delta E < 0$, E_0 will move away from E_1 which corresponds to a blue shift. Hence, using the definition of $\Delta E \equiv \Delta E_0 - \Delta E_1$, the first and second terms in Equation (6) should be both positive, i.e.

$$\Delta\alpha = \alpha(E+\Delta E) - \alpha(E) \approx \frac{1}{2}\Delta p F^2 \frac{\partial \alpha}{\partial E} + \frac{1}{6}(\Delta u \cdot F)^2 \frac{\partial^2 \alpha}{\partial E^2} \tag{7}$$

Experimentally, as shown in Supplementary Figure 4, it is also evident that both the first and second terms should be at the same sign in order to achieve a reasonable fitting result. In brief, the definition of ΔE is determined by the definition of α under electrical field, i.e. $\alpha(E+\Delta E)$. If α under an electric field is expressed as $\alpha(E-\Delta E)$, then ΔE should be defined as $\Delta E \equiv \Delta E_1 - \Delta E_0$, and the final expression of the fitting equation would be the same as in Equation (7).

Supplementary Note 3. Interpretation on the optical effect in Generalized EM fitting equation

If multiple reflection within device is neglected, as can be seen from Equation (3) and Equation (4) in the manuscript, $\frac{\partial R}{R\partial\alpha} = -2d$ or $\frac{\partial T}{T\partial\alpha} = -d$ for R mode and T mode, respectively, which is a constant for a certain active layer thickness d in device. It implies that the EM spectra is totally determined by derivation of optical constant of materials. However, in the generalized model, $\frac{\partial I}{I\partial\alpha}$ and $\frac{\partial I}{I\partial n}$ is no longer a constant and varies as a function of photon energy. Thus, $\frac{\partial I}{I\partial\alpha}$ and $\frac{\partial I}{I\partial n}$ describes optical effect which alters the EM spectra without optical effect which is described by the term in the brackets in Equation (20) in manuscript.

Supplementary Note 4. Study on the contribution from electrorefraction and electroabsorption to EM Spectra

In order to unlock the mechanism behind what makes T mode EM suffers less from electrorefraction effect, optical simulation was conducted to calculate relative contribution from electrorefraction and electroabsorption by defining a dimensionless ratio $\left|\frac{\partial I}{\partial n}:\frac{\partial I}{\partial k}\right|$, where n k value represents the optical constant of active layer. The larger this ratio the stronger electrorefraction will be contributed to EM signal. Supplementary Figure 10 (a) and (b) respectively shows R mode and T mode contour plot of $\left|\frac{\partial I}{\partial n}:\frac{\partial I}{\partial k}\right|$.

As a case study, the device configuration is constructed as ITO(110 nm)/active layer(d nm) /Al(200 nm) for reflection mode and ITO(110 nm)/active layer(d nm) /Ag(16 nm) for T mode. We take 400nm as the probing wavelength. Three points can be summarized from Supplementary Figure 10: 1. It can be seen that for both R and T mode, as the active layer thickness d equals to the thinnest 10 nm, the ratio is over 50% for most n k values; 2. For R mode, from 10nm to 40nm, the blue area which corresponds to smaller electrorefraction ratio firstly increases; and then from 200nm to 2000nm, the blue area decreases and remains almost the same for thickness thicker than 1000nm; 3. For T mode, as active layer thickness becomes thicker, it is intriguing to note that the ratio keeps getting smaller for most k>0 region. Such interesting result of T mode points to the hypothesis that the ratio $\left|\frac{\partial I}{\partial n}:\frac{\partial I}{\partial k}\right|$ is correlated to the thickness of active layer. We establish a series analysis approach to rationalize the above hypothesis. Let us consider multiple reflection which is draw in Supplementary Figure 12. We define the incident light power as 1, the power transmission ratio of air-ITO glass or ITO glass-air is denoted as T_1 , T_2 is defined as metal electrode-air power transmission rate. R_{surf} is the left 1st reflected light beam power from ITO/active layer interface and it is independent of active layer thickness; R_1 is power reflection rate at active layer/ITO glass interface and R_2 is power reflection rate at active layer/metal electrode interface. Thus, the individual reflected light beam power from the left to right can be expressed as series S(1):

$$R_{surf}, T_1^2 R_2 e^{-2\alpha d}, T_1^2 R_1 R_2^2 e^{-4\alpha d}, T_1^2 R_1^2 R_2^3 e^{-6\alpha d}, \ldots . T_1^2 R_1^s R_2^{s+1} e^{-2(s+1)\alpha d} \ldots . \quad \text{S(1)}$$

Similarly, each transmitted light beam power from the left to right can be written as series (S2):

$$T_1T_2e^{-\alpha d}, T_1T_2R_2R_1e^{-3\alpha d}, T_1T_2{R_2}^2{R_1}^2e^{-5\alpha d}\ldots T_1T_2{R_2}^s{R_1}^se^{-(2s+1)\alpha d}\ldots \tag{S2}$$

Where s is an integer number, d is active layer thickness and α is the absorption coefficient of active layer. ${T_1}^2{R_1}^sR_2^{s+1}e^{-2(s+1)\alpha d}$ stands for the general term for individual reflected light beam except for the first beam; $T_1T_2{R_2}^s{R_1}^se^{-(2s+1)\alpha d}$ represents the general term for each individual transmitted light beam. Hence, with simple mathematical calculation, the ratio $\left|\frac{\partial I}{\partial n}:\frac{\partial I}{\partial k}\right|$ of the general term of reflected light beam and transmitted light beam can be expressed as Equation (8) and Equation (9).

$$\begin{aligned}\left|\frac{\partial R}{\partial n}:\frac{\partial R}{\partial k}\right| &= \left|e^{-2(s+1)\alpha d}\frac{\partial {T_1}^2{R_1}^sR_2^{s+1}}{\partial n}:\left(e^{-2(s+1)\alpha d}\frac{\partial {T_1}^2{R_1}^sR_2^{s+1}}{\partial k}-{T_1}^2{R_1}^sR_2^{s+1}e^{-2(s+1)\alpha d}2(s+1)\frac{4\pi}{\lambda}d\right)\right| \\ &= \left|\frac{\partial {T_1}^2{R_1}^sR_2^{s+1}}{\partial n}:\left(\frac{\partial {T_1}^2{R_1}^sR_2^{s+1}}{\partial k}-{T_1}^2{R_1}^sR_2^{s+1}2(s+1)\frac{4\pi}{\lambda}d\right)\right|\end{aligned} \tag{8}$$

$$\begin{aligned}\left|\frac{\partial T}{\partial n}:\frac{\partial T}{\partial k}\right| &= \left|e^{-(2s+1)\alpha d}\frac{\partial T_1T_2{R_2}^s{R_1}^s}{\partial n}:\left(e^{-(2s+1)\alpha d}\frac{\partial T_1T_2{R_2}^s{R_1}^s}{\partial k}-T_1T_2{R_2}^s{R_1}^se^{-(2s+1)\alpha d}(2s+1)\frac{4\pi}{\lambda}d\right)\right| \\ &= \left|\frac{\partial T_1T_2{R_2}^s{R_1}^s}{\partial n}:\left(\frac{\partial T_1T_2{R_2}^s{R_1}^s}{\partial k}-T_1T_2{R_2}^s{R_1}^s(2s+1)\frac{4\pi}{\lambda}d\right)\right|\end{aligned} \tag{9}$$

Considering that for each beam of light if the k value increases, the absorption of active layer increases so that the transmitted or reflected light beam power should decrease. Hence $\frac{\partial R}{\partial k}<0$ and $\frac{\partial T}{\partial k}<0$. When thickness of active layer approaches zero, in order to keep $\frac{\partial R}{\partial k}$ or $\frac{\partial T}{\partial k}$ negative, it is concluded that the term $\frac{\partial {T_1}^2{R_1}^sR_2^{s+1}}{\partial k}<0$ in Equation (8) and $\frac{\partial T_1T_2{R_2}^s{R_1}^s}{\partial k}<0$ in Equation (9). So as the active layer thickness gets thicker, the

absolute value of $\frac{\partial R}{\partial k}$ and $\frac{\partial T}{\partial k}$ increases and the ratio $\left|\frac{\partial I}{\partial n}:\frac{\partial I}{\partial k}\right|$ should keep decreasing which is as expected for the general trend of the ratio in T mode as shown in Supplementary Figure 13(b) and is also as expected for the general trend of the $\left|\frac{\partial I}{\partial n}:\frac{\partial I}{\partial k}\right|$ in R mode when the active layer thickness is less than 200nm as shown in Supplementary Figure 13(a). However, for R mode as the active layer is thick enough, R_{surf} in series (S1) should dominant which is independent of active layer thickness and the rest terms signal is too weak to be detected. So when the active layer thickness exceeds a certain value the whole $\left|\frac{\partial I}{\partial n}:\frac{\partial I}{\partial k}\right|$ profile should gradually resemble the one when active layer is thick enough and the light intensity reflected by the active layer/metal cathode interface can be neglected, and this is consistent with the profile trend as d >200nm in Supplementary Figure 13(a). While for T mode, as the active layer is thick enough, only the 1st term in series (S2) is taken into account which corresponds to s=0 in Equation (9) and $\frac{\partial T}{\partial k}$ keeps increasing as active layer thickness increases and this is consistent with the trend as shown in Figure 13(b).

Supplementary References:

1. C. Huber et al. Electroreflectance of thin-film solar cells: Simulation and experiment. *Phys. Rev. B* **92**, 075201 (2015).
2. V. Roiati et al. Stark effect in perovskite/TiO2 solar cells: evidence of local interfacial order. *Nano. Lett.* **14**, 2168-2174 (2014).
3. P. J. Brewer et al. Internal Field Screening in Polymer Light-Emitting Diodes. *Adv. Funct. Mater.* **14**, 562-570 (2004).
4. E. Amerling et al. Electroabsorption Spectroscopy Studies of (C4H9NH3)2PbI4 Organic-Inorganic Hybrid Perovskite Multiple Quantum Wells. *J. Phys. Chem. Lett.* **8**, 4557-4564 (2017).
5. X. Wu et al. Composition-Dependent Light-Induced Dipole Moment Change in Organometal Halide Perovskites. *J. Phys. Chem. C* **119**, 1253-1259 (2015).
6. P. A. Lane et al. Electroabsorption studies of phthalocyanine-perylene solar cells. *Sol. Energy. Mat. Sol. C* **63**, 3-13 (2000).
7. S. J. Martin, D. D. C. Bradley, P. A. Lane, and H. Mellor. Linear and nonlinear optical properties of the conjugated polymers PPV and MEH-PPV. *Phys. Rev. B* **59**, 15133 (1999).
8. S. J. M. Liess, Z. V. Vardeny,M. Ozaki,K. Yoshino,Y. Ding, and T. Barton. Electroabsorption spectroscopy of luminescent and nonluminescent p-conjugated polymers. *Phys. Rev. B* **56**, 15712-15723 (1997).
9. L. Sebastain, G. Weiser, and H. Bassler. Charge transfer transitions in solid tetracene and pentacene studied by electroabsorption. *Chem. Phys.* **61**, 125-135 (1981).
10. B. Bernardo et al. Delocalization and dielectric screening of charge transfer states in organic photovoltaic cells. *Nat. Commun.* **5**, 4245 (2014).
11. S. J. Martin, H. Mellor, Donal DC. Bradley, and P. L. Burn. Electroabsorption studies of PPV and MEHPPV. *Opt. Mat.* **9**, 88-93 (1998).
12. G. U. Bublitz, and S. G. Boxer. Stark spectroscopy: applications in chemistry, biology, and materials science. *Annu. Rev. Phys. Chem.* **48**, 213 (1997).
13. S. Kazaoui et al. Comprehensive analysis of intermolecular charge-transfer excited states in C_{60} and C_{70} films. *Phys. Rev. B* **58**, 7689 (1998).
14. F. W. Vance, R. D. Williams, and J. T. Hupp. Electroabsorption spectroscopy of molecular inorganic compounds. *Int. Rev. Phys. Chem.* **17**, 307 (1998).
15. L. Sebastian, G. Weiser, G. Peter, and H. Bassler. charge transfer transitions in crystalline anthracene. *Chem. Phys.* **75**, 103 (1983).
16. F. Feller, and A. P. Monkman. Electroabsorption studies of poly2,5-pyridinediy. *Phys. Rev. B* **60**, 8111-8116 (1999).
